# Genetic Variants of *CLPP* and *M1AP* Are Associated With Risk of Non-Small Cell Lung Cancer

**DOI:** 10.3389/fonc.2021.709829

**Published:** 2021-09-15

**Authors:** Xianghan Li, Yiran Zou, Teng Li, Thomas K. F. Wong, Ryan T. Bushey, Michael J. Campa, Elizabeth B. Gottlin, Hongliang Liu, Qingyi Wei, Allen Rodrigo, Edward F. Patz

**Affiliations:** ^1^Research School of Biology, Australian National University, Canberra, ACT, Australia; ^2^School of Biological Sciences, University of Auckland, Auckland, New Zealand; ^3^Department of Radiology, Duke University Medical Center, Durham, NC, United States; ^4^Duke Cancer Institute, Duke University Medical Center, Durham, NC, United States; ^5^Department of Population Health Sciences, Duke University School of Medicine, Durham, NC, United States; ^6^Department of Medicine, Duke University School of Medicine, Durham, NC, United States; ^7^Department of Pharmacology and Cancer Biology, Duke University Medical Center, Durham, NC, United States

**Keywords:** non-small cell lung cancer, single nucleotide polymorphism, lung cancer risk, *CLPP*, *M1AP*

## Abstract

**Background:**

Single nucleotide polymorphisms (SNPs) are often associated with distinct phenotypes in cancer. The present study investigated associations of cancer risk and outcomes with SNPs discovered by whole exome sequencing of normal lung tissue DNA of 15 non-small cell lung cancer (NSCLC) patients, 10 early stage and 5 advanced stage.

**Methods:**

DNA extracted from normal lung tissue of the 15 NSCLC patients was subjected to whole genome amplification and sequencing and analyzed for the occurrence of SNPs. The association of SNPs with the risk of lung cancer and survival was surveyed using the OncoArray study dataset of 85,716 patients (29,266 cases and 56,450 cancer-free controls) and the Prostate, Lung, Colorectal and Ovarian study subset of 1,175 lung cancer patients.

**Results:**

We identified 4 SNPs exclusive to the 5 patients with advanced stage NSCLC: rs10420388 and rs10418574 in the *CLPP* gene, and rs11126435 and rs2021725 in the *M1AP* gene. The variant alleles G of SNP rs10420388 and A of SNP rs10418574 in the *CLPP* gene were associated with increased risk of squamous cell carcinoma (OR = 1.07 and 1.07; P = 0.013 and 0.016, respectively). The variant allele T of SNP rs11126435 in the *M1AP* gene was associated with decreased risk of adenocarcinoma (OR = 0.95; P = 0.027). There was no significant association of these SNPs with the overall survival of lung cancer patients (P > 0.05).

**Conclusions:**

SNPs identified in the *CLPP* and *M1AP* genes may be useful in risk prediction models for lung cancer. The previously established association of the *CLPP* gene with cancer progression lends relevance to our findings.

## Introduction

Lung cancer is the leading cause of cancer death in the world with 2 million new cases and 1.7 million deaths in 2018 (1). In the United States, 228,820 new cases of lung cancer and 135,720 deaths were projected to occur in 2020 (2). The majority (~85%) of lung cancer cases are non-small cell lung cancer (NSCLC), and the overall 5-year survival rate is only ~20%. New targeted and immune modulating therapies are expected to improve survival rates, but it often remains unclear which patients will respond and are at high risk for recurrence. Since genetic predisposition plays a role in tumor phenotype, the identification of risk, predictive, and prognostic markers may guide clinical decisions (3).

The current study initially began as an exploration of tumor cell evolution as a method of developing a prognostic biomarker for NSCLC. Tumor growth and metastasis can be viewed in an evolutionary context (4, 5). The clonal heterogeneity of tumors is a reflection of environmental pressures on a genetically diverse population of cells. As part of this study, we examined the most appropriate germ line reference standard against which to search for cancer mutations, and unexpectedly discovered four single nucleotide polymorphisms (SNPs) from resected normal lung tissue in patients with advanced stage NSCLC (n = 5) that were not found in patients with early stage disease (n = 10). We then determined in independent validation datasets (OncoArray and PLCO databases) that two of the four SNPs are associated with a higher risk of developing squamous cell carcinoma and one of the four SNPs was associated with a lower risk of developing adenocarcinoma. Despite these SNPs being identified in advanced stage patients, however, we did not find an association with survival.

## Methods

### Discovery Patients and Samples

Duke University follows the set of ethical principles outlined in “The Belmont Report: Ethical Principles and Guidelines for the Protection of Human Subjects of Research”. The Duke University Health System Institutional Review Board approved this study and all subjects gave written informed consent for the use of their tissue in this study. Fifteen sequential NSCLC patients were chosen for this study. These patients are described in **Table 1**. The 10 patients with early stage NSCLC had a post-resection recurrence-free duration ranging from 14-45 months. The duration endpoint for the non-metastatic designation in each of these cases was the last time they were examined in the clinic following initial diagnosis. Five other patients had metastasis at presentation: in four cases, metastasis to a lymph node, in one case, to the lung.

**Table 1:** Study patients.

Patient ID	Sex	Age at DX	Stage at DX	Histology	Status	For NM, recurrence-free duration, mo.
16011	F	46	IB	AC	NM	45
16028	M	71	IIB	SCC	M to LN	
16030	F	73	IIIA	SCC	M to LN	
17004	F	78	IA	SCC	NM	40
17005	M	76	IA	SCC	NM	32
17008	F	56	IA	AC	NM	18
17011	M	69	IB	AC	NM	14
17012	M	80	IIA	AC	NM	15
17014	F	75	IA	AC	NM	37
17015	M	74	IB	LCC	NM	19
17017	F	66	IIA	LCC	M to LN	
17028	F	72	IA	AC	NM	27
17029	M	64	IV	SpCC	M to lung	
17030	M	61	IB	AC	NM	36
18001	F	79	IIB	AC	M to LN	

Patients indicated in red were diagnosed with metastatic NSCLC. AC, adenocarcinoma; SCC, squamous cell carcinoma; LCC, large cell carcinoma; SpCC, spindle cell carcinoma; NM, non-metastatic NSCLC; M, metastatic NSCLC; LN, lymph node; DX, diagnosis; and for sex, M, male; F, female. Recurrence-free duration for the NM patients is as of their last clinic visit.

### Isolation of DNA From Normal Lung Tissue

Normal lung DNA was isolated from lung tissue that had been obtained at surgery and stored at −80°C or from FFPE sections using either a QIAamp Fast DNA Tissue kit or a QIAamp DNA FFPE Tissue kit (Qiagen; Hilden, Germany), respectively, according to the manufacturer’s recommendations.

### Whole Genome Amplification

Whole genome amplification (WGA) was carried out using a REPLI-g Single Cell kit (Qiagen). This kit, which was used for bulk DNA amplification from normal lung tissue, uses multiple displacement amplification (MDA) technology (6). Prior to exome sequencing, WGA was verified by PCR amplification of a region of the kinesin light chain 2 gene (KCL2) followed by agarose gel electrophoresis.

### Whole Exome Sequencing

Whole genome amplified lung DNA was first treated with KAPA Beads (Roche; Basel, Switzerland) to remove any residual buffer or EDTA prior to library preparation. Sequencing libraries were then created using KAPA HyperPLUS reagents (Roche) on the Hamilton NGS Star liquid handler (Hamilton Robotics; Cary, NC, USA) according to the manufacturer’s instructions. Briefly, samples were enzymatically fragmented, end repaired, A-tailed, and ligated to sample barcodes (Integrated DNA Technologies [IDT]; Coralville, IA, USA). Following library preparation and amplification, libraries were normalized and pooled for exome capture. Sequence capture was performed on the Hamilton NGS Star liquid handler using the IDT xGen Exome Research panel according to the rapid capture protocol. Captured libraries were visualized on the AATI Fragment Analyzer (Agilent Technologies; Santa Clara, CA, USA) for quality. Finally, samples were quantitated by qPCR with KAPA Quant (Roche) and normalized prior to pooling and sequencing on the Illumina NovaSeq 6000 sequencer (Illumina, Inc.; San Diego, CA, USA).

### Data Pre-Processing

For every sample, sequencing reads were aligned to the human reference genome (hs37d5) using BWA MEM (7) as implemented in the Sentieon Genomics bioinformatics suite (8). Reads with a mapping quality score less than 40 were discarded. Duplicated reads in the alignment were removed using SAMtools (7). The alignment was further indel-realigned based on known indels from the 1000 Genomes Project (9), while the base quality scores were then recalibrated based on known indels from the 1000 Genomes Project (9) and SNPs from dbSNP (10), both using GATK (11) in Sentieon Genomics (8). To evaluate the quality of sequencing data, the mean coverage of each sequence on the targeted exome regions was measured using BED tools (12). The target region was downloaded from the xGen Exome Research Panel website (https://sg.idtdna.com/pages/products/next-generation-sequencing/hybridization-capture/lockdown-panels/xgen-exome-research-panel).

### SNP Detection From Normal Bulk Sequences

Varscan2 (13) and Haplotype Caller of GATK (11) with default parameters were used to detect single nucleotide polymorphisms (SNPs) from normal bulk sequences. The following command line setting was used with VarScan2: samtools mpileup -B -f reference.fasta myData.bam | java -jar VarScan.v2.2.jar mpileup2snp.

For GATK, the recommended pipeline “Germline SNVs + Indels” given in the link https://software.broadinstitute.org/gatk/best-practices/workflow?id=11145 was used.

Only SNPs detected by both Varscan2 and Haplotype Caller were retained to avoid false-positive variant calls. Each SNP inside the VCF file was subjected to further filtering to require having at least 10 supported reads and had coverage across all bulk sequences. The filtered VCF file was annotated using ANNOVAR (14). After annotation, the SNPs that differentiated the metastatic patients from non-metastatic patients were selected.

### Independent Validation of SNPs

We investigated the associations of the identified SNPs with the risk of lung cancer as well as the survival of lung cancer patients by using the available data from OncoArray and PLCO, respectively (15–18).

The GWAS data of OncoArray were requested from dbGAP (dbGAP accession #: phs001273.v3.p2), in which genotyping was performed with Illumina Infinium OncoArray-500k and imputation was performed with IMPUTE2 with the reference dataset of 1000 Genomes Project Phase 3 (Haplotype release date October 2014) (18). The OncoArray dataset contained data derived from the germline DNA sequences of 85,716 patients (29,266 cases and 56,450 cancer-free controls). Logistic regression was used to assess the associations between SNPs and lung cancer risk in each study with adjustment for the top significant principal components. Fixed-effects meta-analysis was used to combine the results of different studies if the Cochran’s Q-test P > 0.100 and the heterogeneity statistic (I2) < 50%. Otherwise, a random-effects model was applied.

For survival analysis, there were 1,185 NSCLC patients from PLCO with both survival and GWAS data available. The GWAS dataset was requested from dbGaP (the approval project number: PLCO-95), for which genomic DNA samples extracted from the whole blood were genotyped with Illumina HumanHap240Sv1.0, HumanHap300v1.1 and HumanHap550v3.0 (dbGaP accession #: phs000093.v2.p2 and phs000336.v1.p1). Imputation was performed with ±500kb flanking buffer regions around the targeted SNPs using miniMac4 and the reference panel of the European data from the 1,000 Genomes Project (phase 3). We then performed a Cox proportional hazards regression analysis to evaluate associations between each of the SNPs and overall survival (in an additive model) with adjustment for age, sex, smoking status, histology, tumor stage, chemotherapy, radiotherapy, surgery and the first four principal components of the population structures in the genotyping data. The distribution of the applied clinical variables can be found in the previous publication (19).

### Functional Annotation and Predictive Analysis

To further explore the functional associations of the four SNPs examined in this study (rs11126435, rs2021725, rs10420388 and rs10418574), we annotated non-coding variants using ANNOVAR (14). To analyze the predicted functional effects of these variants, we used several web tools. PredictSNP2 (https://loschmidt.chemi.muni.cz/predictsnp2/) was used to identify potential functional effects of the SNPs (20). HaploReg (http://pubs.broadinstitute.org/mammals/haploreg/haploreg.php) was used to identify SNPs that are in linkage disequilibrium (LD) with the SNPs of interest in order to shed light on their potential regulatory effects (21). The Genotype-Tissue Expression (GTEx) project portal (https://gtexportal.org/home/) was used to investigate relevant expression quantitative trait loci (eQTLs) for the four SNPs in lung tissue (22). RegulomeDB 2.0 (https://regulomedb.org/regulome-search/) was used to estimate the functional relevance of the SNPs. RegulomeDB 2.0 ranks the possibility of the SNP to be a regulatory element from 1a (most likely) to 6 (minimal binding evidence) (23).

## Results

### Detection of SNPs That Differentiate Between Metastatic and Non-Metastatic Groups

We performed whole exome sequencing of bulk normal lung DNA from 15 NSCLC patients, 10 with early stage, non-metastatic disease and 5 with advanced stage disease. Each bulk sequence had >100× mean sequencing depth.

In exome regions, there were no SNPs that differentiated the two groups of patients, while in intron regions, four SNPs differentiated the two groups (**Table 2**). Two intron variants (rs11126435 and rs2021725) belong to the meiosis 1 associated protein (*M1AP*) gene. Two intron variants (rs10420388 and rs10418574) belong to the caseinolytic mitochondrial matrix peptidase proteolytic subunit (*CLPP*) gene. None of the early stage patients had polymorphisms at those four sites, while all the metastatic patients were heterozygous at all the sites. The SNP genotypes for each patient are shown in **Table 3**.

**Table 2:** SNPs that differentiate between groups.

SNP ID	Reference (hs37d5)	Genotype		Gene: consequence
		NM	M	
rs11126435	A/A	A/A	A/T	*M1AP*: intron variant
rs2021725	A/A	A/A	A/G	*M1AP*: intron variant
rs10420388	A/A	A/A	A/G	*CLPP*: intron variant
rs10418574	G/G	G/G	G/A	*CLPP*: intron variant

“NM” indicates genotype in non-metastatic patients. “M” indicates genotype in metastatic patients. The supported reads for each patient are presented in **Supplementary Table 1**.

**Table 3:** SNP genotypes for each NSCLC patient.

Patient ID	Status	*M1AP*		*CLPP*	
		SNPrs11126435	SNPrs2021725	SNPrs10420388	SNPrs10418574
16011	NM	No	No	No	No
16028	M	Yes	Yes	Yes	Yes
16030	M	Yes	Yes	Yes	Yes
17004	NM	No	No	No	No
17005	NM	No	No	No	No
17008	NM	No	No	No	No
17011	NM	No	No	No	No
17012	NM	No	No	No	No
17014	NM	No	No	No	No
17015	NM	No	No	No	No
17017	M	Yes	Yes	Yes	Yes
17028	NM	No	No	No	No
17029	M	Yes	Yes	Yes	Yes
17030	NM	No	No	No	No
18001	M	Yes	Yes	Yes	Yes

“NM” indicates genotype in non-metastatic patients. “M” indicates genotype in metastatic patients. “Yes” indicates the patient contains this SNP, while “No” indicates the patient has the same genotype as reference hs37d5.

### Association of SNPs and Survival

Four SNPs were extracted from the imputation data, and their associations with lung cancer risk and survival were assessed in the OncoArray study and the PLCO study. (The rs2021725 SNP was available in the PLCO dataset but not in the OncoArray dataset.) As shown in **Supplementary Table 2**, we found that the variant alleles G and A of the two SNPs (rs10420388 and rs10418574) in *CLPP* were associated with an increased risk of squamous cell carcinoma (OR = 1.07 and 1.07; *P* = 0.013 and 0.016, respectively), while the variant allele T of SNP rs11126435 in *M1AP* was associated with a decreased risk of adenocarcinoma (OR = 0.95; *P* = 0.027). However, no significance was found for these SNPs in association with the overall survival of lung cancer patients, either in the overall analysis or stratified analysis by stage and histological type (*P* > 0.05; **Supplementary Table 3**).

### Functional Annotation and Predictive Analysis

ANNOVAR was used to determine whether non-coding variants rs11126435, rs2021725, rs10420388 and rs10418574 may disrupt regulatory elements in the human genome. The results show that *M1AP* variants rs11126435 and rs2021725 fall in a weak enhancer unit, while *CLPP* variants rs10420388 and rs10418574 fall in a transcriptional elongation unit. PredictSNP2 predicts that all four variants have a neutral influence on regulatory function (**Supplementary Table 4**).

HaploReg was used to find SNPs in LD with the SNPs of interest and provide clues to their possible regulatory effects. There are 33 SNPs in LD (r^2^ > 0.8) with *CLPP* variants rs10420388 and rs10418574 (**Supplementary Table 5**). These SNPs affect transcription factor (TF) binding sites, either promoters or enhancers.

There are 9 SNPs in LD (r^2^ > 0.8) with *M1AP* variant rs11126435. These SNPs affect TF binding. There are 13 SNPs in LD (r^2^ > 0.8) with *M1AP* variant rs2021725. Most of these SNPs are histone marks for promoters and enhancers and thus would also be indicative of sites of TF binding.

In the GTEx portal (https://gtexportal.org/home/), the *M1AP* variants rs11126435 and rs2021725 are both inferred to be eQTLs for *LBX2*, *LBX2-AS1*, *INO80B*, *RP11-523H20.3* and *MOGS* genes (P-values ranging from 0.000016 to 1.70×10^-13^), indicating the two SNPs are associated with the expression level of these five genes in lung tissue (**Supplementary Table 6**). It should be noted that, among these five genes, *LBX2* and *LBX2-AS1* genes have been found to be related to lung cancer development (24, 25). The *M1AP* variants rs11126435 and rs2021725 are not inferred to be eQTLs for the *M1AP* gene in the GTEx portal.

GTEx predicts that both *CLPP* variants rs10418574 and rs10420388 are eQTLs for *GTF2F1* and *CLPP* genes in lung tissue (P-values ranging from 0.000001 to 5.00×10^-7^), suggesting that the two SNPs are relevant to the expression level of *GTF2F1* and *CLPP* genes.

For *M1AP* variant rs11126435, RegulomeDB 2.0 shows a rank of 1b, indicating a high probability of functional relevance for this SNP. For *M1AP* variant rs2021725 and *CLPP* variant rs10420388, RegulomeDB 2.0 scores are 1f and 2b, respectively, indicating a likely probability of functionality for each of these two SNPs. *CLPP* variant rs10418574 has a rank of 4 in RegulomeDB 2.0, suggesting minimal functionality for this SNP (**Supplementary Table 7**).

## Discussion

Lung cancer remains a significant worldwide public health problem, and improvements in early detection and therapy are clearly needed to improve outcomes. The genome-wide association study (GWAS) is an effective method of detecting SNPs, typically using Illumina genotyping platforms (26). This approach identifies SNPs at the genome level, which may be very useful in identifying biomarkers and relevant therapeutic targets. To determine whether the SNP locus is associated with cancer, the allelic frequency of the SNP marker has to significantly differ between the cancer group and the control group (26).

To date, GWAS have successfully identified susceptibility loci associated with lung cancer (27). Moreover, to investigate further biological functions of the associated SNPs, the analysis strategies have moved from single-marker analyses to pathway-based analyses (27). However, most GWAS studies focus on the statistical and biological significance of individual SNPs. In the present study, for each patient, we traced the genetic changes of four SNP sites in patients with different NSCLC phenotypes: early stage, non-metastatic or metastatic. Note that the SNPs were found in intron regions despite our having used exome sequencing technology. A previous study illustrated that exome sequencing can also produce high-quality sequence outside the target region (28). Hence, those intronic SNPs were considered in our analysis.

In the present study, we discovered 4 SNPs from normal lung tissue of patients with advanced stage lung cancer, and then explored if they were associated with increased risk of developing the disease and survival in independent validation datasets. These types of biomarkers could complement current risk prediction models and potentially help guide therapeutic options. Lung cancer risk was assessed for rs10420388 and rs10418574 in *CLPP* and rs11126435 in *M1AP* in the OncoArray study. (*M1AP* rs2021725 was not available in this dataset.) Notably, patients with rs10420388 and rs10418574 in *CLPP* showed a significantly increased risk of squamous cell carcinoma, while rs11126435 in *M1AP* was associated with decreased risk of adenocarcinoma. Thus, in clinical practice these SNPs could be used, most likely in combination with other SNPs or markers, to more efficiently determine which patients may benefit from more intense screening programs. This is currently an area of intense interest. To our knowledge, this is the first report that *M1AP* rs11126435, *CLPP* rs10420388 and *CLPP* rs10418574 variant alleles may be associated with an NSCLC risk phenotype. However, we did not find an association of any of the 4 SNPs with survival, despite the fact that the SNPs were identified in patients with advanced stage disease and presumably worse outcomes.

*M1AP*, located on chromosome 2, encodes meiosis 1 associated/arrest protein that is likely to function in the progression of meiosis. Previous studies demonstrated that mutations in *M1AP* were associated with oligozoospermia (29, 30). A retroviral insertion mutagenesis study showed that *M1AP* synergized with *Cbfb* (core-binding factor)-*MYH11* (myosin, heavy chain 11) translocation during the onset of acute myeloid leukemia (31). However, the role of *M1AP* in cancer progression remains unclear. The two SNPs detected in the intron region of *M1AP* are not inferred to be eQTLs for *M1AP* itself. Although they are not predicted to be associated with expression level of *M1AP*, these two SNPs are predicted as eQTLs for two lung cancer-related genes: *LBX2*, encoding a transcription factor causally implicated in lung adenocarcinoma (24) and *LBX2-AS1* (25), encoding a regulatory RNA that promotes cell proliferation and metastasis of NSCLC. The two adjacent genes are located approximately 60 and 72 kilobases from rs11126435 and rs2021725, respectively. Therefore, these SNPs may affect the expression of one or both of *LBX2* and *LBX2-AS* and not *M1AP*.

*CLPP* is a protein-coding gene located on chromosome 19 (32). The protein encoded by *CLPP* is a serine protease associated with the inner mitochondrial membrane that degrades misfolded proteins and is required for mitochondrial respiration (33–36). The CLpP protein is overexpressed in many human cancers, including NSCLC regardless of histotype, and is most highly expressed in primary NSCLC lesions that developed metastasis (36). In several types of cancer (including breast, lung, and uveal cancer), overexpressed CLpP is associated with shorter metastasis-free survival (36). It was also shown that CLpP supports tumor cell proliferation and motility (33, 36). Gene sequencing plus predictive functional analyses indicate that the rs10420388 and rs10418574 *CLPP* variants are associated with intronic elements involved in transcriptional regulation. Therefore, we propose that these variants may cause overexpression of the *CLPP* gene.

As noted above, this study was designed as a pilot project to examine the evolutionary dynamics of tumor cells in patients with early stage vs. late stage disease. As such, the identification of SNPs reported here is an incidental outcome of a study that was not, in itself, focused on identifying either prognostic markers or causal genetic mechanisms. This means, among other things, that there may be (indeed, are likely to be) more SNPs that have not been identified in our study. However, the small sample size in this study and the variability of coverage also means that we have low statistical power to identify SNPs that may be good markers. Because it is likely that we have not characterized the constellation of SNPs that may separate the two groups of patients, it is difficult to construct a hypothesis about the functional and regulatory pathways that steer individuals with NSCLC into metastatic or non-metastatic progression.

It is also worth noting that the GWAS datasets of OncoArray and PLCO used to validate the markers we identified in this study only include Caucasian populations. Thus, our results may not be generalizable to other ethnic groups, and further studies are needed to determine the true impact on lung cancer risk in the general population.

Because cancer is a disease of altered genes, GWAS and identification of SNPs offers an important strategy to studying cancer risk and biologic features that may be essential for tumor development and progression. In the current study we focused on 4 SNPs discovered from the normal lung of patients with advanced stage NSCLC as compared to early stage disease and showed that three of these markers were associated with lung cancer risk. The exact molecular mechanisms by which they influence cancer cells remain unclear, and although these data may be useful in risk-prediction models, further biochemical studies and functional experiments are warranted.

## Data Availability Statement

The datasets presented in this study can be found in the NCBI Bioproject repository under accession number PRJNA750918; link: https://www.ncbi.nlm.nih.gov/bioproject/PRJNA750918.

## Ethics Statement

The studies involving human participants were reviewed and approved by Duke University Health System Institutional Review Board. The patients/participants provided their written informed consent to participate in this study.

## Author Contributions

AR, QW, and EP conceived of and designed the study and performed supervision. MC and EG selected the patients for the study and followed their cancer statuses. MC and RB performed DNA manipulations. XL, YZ, TL, TW, and HL curated and analyzed the data. All authors contributed to the article and approved the submitted version.

## Funding

This work was funded by Department of Defense Idea Award W81XWH-15-1-0243 to EP.

## Conflict of Interest

The authors declare that the research was conducted in the absence of any commercial or financial relationships that could be construed as a potential conflict of interest.

## Publisher’s Note

All claims expressed in this article are solely those of the authors and do not necessarily represent those of their affiliated organizations, or those of the publisher, the editors and the reviewers. Any product that may be evaluated in this article, or claim that may be made by its manufacturer, is not guaranteed or endorsed by the publisher.
